# Method optimization and validation for the simultaneous determination of arachidonic acid metabolites in exhaled breath condensate by liquid chromatography-electrospray ionization tandem mass spectrometry

**DOI:** 10.1186/1745-6673-1-5

**Published:** 2006-05-17

**Authors:** Luis M Gonzalez-Reche, Anita K Musiol, Alice Müller-Lux, Thomas Kraus, Thomas Göen

**Affiliations:** 1 Institute and Outpatient-Clinic for Occupational and Social Medicine, University Hospital, Aachen University of Technology, Pauwelsstrasse 30, D-52074 Aachen, Germany; 2 Institute for Occupational, Social and Environmental Medicine, University Erlangen-Nuremberg, Schillerstr. 29, D-91054 Erlangen, Germany

## Abstract

**Background:**

Determinations of inflammatory markers in exhaled breath condensate were used to assess airway inflammation. The most applied method for this kind of determination is enzyme immunoassay. For research purposes to find new or to relate concrete biomarkers to different pulmonary diseases, a simultaneous determination of different inflammatory markers would be advantageous.

**Methods:**

We developed an analytical method with on-line clean up and enrichment steps to determine 12 different inflammatory markers in exhaled breath condensate. A specific detection method ensures the unequivocally determination of each analyte at the same run. The method was optimized and validated to achieve a low limit of quantification up to 10 pg/mL each analyte. The precision of the method ranged between 4 and 16%.

**Conclusion:**

The presented method should serve as an easy and fast tool to assess the utility of inflammatory markers in exhaled breath condensate to different pulmonary diseases and for several related disciplines in medicine.

## Background

Different markers in exhaled breath condensate (EBC) have been measured and used for the assessment and monitoring of airway inflammation [1]. Airway inflammation is a consequence of many lung diseases such as asthma, cystic fibrosis or chronic obstructive pulmonary diseases (COPD) [2-4]. In occupational medicine, many problems arise from allergic reactions related with pulmonary diseases, which should be assessed for further medical proceedings. Analysis of EBC is a non invasive method for the measurement of low-volatile inflammatory mediators that are known to be exhaled with the expired water vapour from individuals [5]. In contrast to invasive techniques such as bronchoalveolar lavage and bronchial biopsies, the EBC sample collection can be used repeated times and does not induce an inflammatory response by itself. Easy non-invasive sample collection is an important task in occupational medicine where workers examination issues are often a voluntary matter.

Eicosanoids are mediators derived from arachidonic acid and include prostaglandins (PG), isoprostanes and leukotrienes (LT). These eicosanoids were used to try to assess the lung inflammation in patients with pulmonary disease. Some prostaglandins and thromboxane could have proinflammatory or anti-inflammatory properties [6]. Leukotrienes are potent constrictors and proinflammatory mediators. Leukotrienes LTC_4_, LTD_4 _and LTE_4 _are known as cysteinyl-leukotrienes [7].

Isoprostanes are formed by free radical-catalyzed lipid peroxidation of arachidonic acid and act as a bioactive product of lipid peroxidation [8]. Their formation is increased by systemic oxidative stress [9]. Studies were conducted to determine 8-isoprostane in EBC of patients with different pulmonary diseases [10-13].

GC/MS [7], LC/MS [14], RIA [15] and ELISA analytical techniques were used for the quantification of this kind of substances in EBC. Determinations of inflammatory markers in EBC with ELISA could only be done for one substance or at best as a sum of parameters.

It was the aim of this study to optimise and validate an analytical procedure to determine simultaneously different inflammatory markers in EBC with a specific detection such as mass spectrometry (MS) in contrast to the mostly applied ELISA analytical methods applied yet. For a sensitive detection, including structural information, tandem mass spectrometry was used to determine unequivocally prostaglandins and leukotrienes. This developed method could serve to monitor inflammatory markers in EBC of workers for further necessary research in occupational medicine.

## Methods

With recent progress in liquid chromatography separations and mass spectrometry detection systems, improvement in sensitivity and simultaneous detection of multiple analytes is possible. However, the determination of these kinds of markers in breath condensate makes a sample enrichment step unavoidable when attempting to achieve a low limit of detection to cover the expected range at the lower pg/mL.

By combining the online enrichment and the LC/MS/MS techniques we have developed an analytical method for the sensitive detection of 12 different inflammatory mediators and oxidative stress markers, trying to make a contribution to the determination of inflammatory marker in EBC to improve and simplify research concerning pulmonary diseases for different disciplines in medicine.

### Chemicals

Prostaglandin D_2 _(PGD_2_), 13,14-dihydro-15-keto-PGD_2_, 11β-PGF_2α_, PGJ_2_, Δ^12^-PGJ_2_, PGF_2α_, 13,14-dihydro-15-keto-PGF_2α_, PGE_2_, 15-keto-PGE_2_, 13,14-dihydro-15-keto-PGE_2_, 8-iso-PGF_2α_, 15-deoxy-Δ^12,14^-PGJ_2_, 6-keto-PGF_1α_, 6,15-diketo-13,14-dihydro-PGF_1α_, the Leukotrienes LTB_4 _and LTE_4_as analytical standards and the labelled [^2^H_4_] LTB_4 _and [^2^H_4_] PGE_2 _as internal standards were purchased from Cayman Chemicals Company (Michigan, USA). All analytical standards had chemical purity >98%.

Acetonitrile was purchased from J.T. Baker (Germany), methanol (GC-grade), acetic acid (glacial, pro analysi) and ammonium acetate p.a. was purchased from Merck (Darmstadt, Germany). Bi-distilled water was used for HPLC mobile phase mixture.

### Standard preparation and internal standardization

A stock solution was prepared containing 10 μg/mL of each described analytes in ammonium acetate 10 mM/methanol 1:1 (v/v). This stock solution was aliquoted and stored at -80°C in 1,5 ml eppendorf reaction tubes until further use. 100 μL of the stock solution was placed in a 100 mL glass volumetric flask and diluted to the mark with ammonium acetate 10 mM obtaining a 10 ng/mL solution. This solution was used as working solution for the preparation of the other standard concentrations for calibration and quality control material.

**Figure 3:**
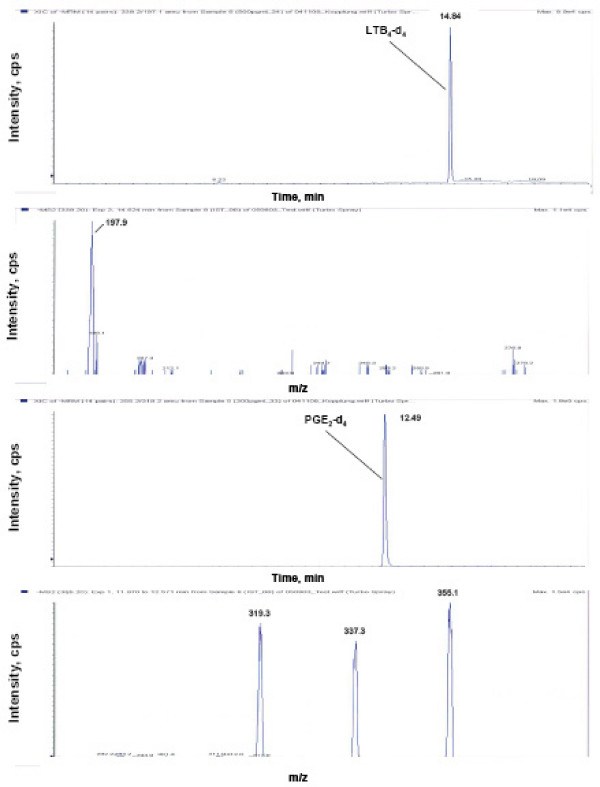
Standard chromatogram of the deuterated standards with the corresponded product ion scans.

For the preparation of internal standards solutions commercially available [^2^H_4_] LTB_4 _and [^2^H_4_] PGE_2 _were used (Figure 3). A stock solution of 100 ng/mL in ammonium acetate 10 mM was prepared. A 1 mL aliquot of the stock solution of the internal standard was placed in a 5 mL glass volumetric flask and diluted to the mark with ammonium acetate 10 mM obtaining a 20 ng/mL solution for each labelled standard. [^2^H_4_] LTB_4 _was used for the correction of leucotriene response values, whereas [^2^H_4_] PGE_2 _was used for prostaglandins and 8-isoprostane. For quantification, the peak area ratio of prostaglandins derivatives analytes to [^2^H_4_] PGE_2 _and the peak area ratio of leukotrienes derivatives to [^2^H_4_] LTB_4 _were used.

### EBC sample collection

The commercial available ECOSCREEN condenser from Viasys-Healthcare (Hoechberg, Germany) was used for the EBC sample collection. The subjects were encouraged to perform tidal breath for 15 minutes through the mouthpiece connected to the condenser while wearing a nose clip. The resulted EBC volumes ranged from 1 to 3 mL. Samples were aliquoted in 1.5 mL Eppendorf microtubes and stored at -80°C until analysis. Detailed description about collection of exhaled breath condensate is described elsewhere [16].

### Sample preparation

Frozen EBC samples/standard solution were thawed and allowed to equilibrate to room temperature. 1 mL aliquots of each sample were transferred to 1.8 mL glass screw-cap vial for HPLC analysis and 100 μL of the working solution of the internal standard were added to each sample. Then the samples were vortex mixed and a 900 μL aliquot was injected into the LC/MS/MS system for quantitative analysis.

### Calibration procedure and quality control

From the working solution of analytical standards described before, six calibration standards in the range from 10 to 500 pg/mL were prepared by diluting the solution with ammonium acetate 10 mM. Linear calibration curves were obtained by plotting the quotients of the peak areas of each analyte with the assigned internal standard [^2^H_4_] LTB_4 _or [^2^H_4_] PGE_2 _as a function of the concentrations used. These calibration curves were used to ascertain the spiked analytes in the EBC samples.

There was no control material commercially available. Therefore quality control material was prepared in the laboratory spiking an ammonium acetate buffer with the corresponded amounts of analytes. Two concentration levels covering the upper and the lower concentration range were prepared for quality control. For the low-concentration quality control material (Q1) we spiked ammonium acetate 10 mM with 50 pg each analyte per mL, whereas for the high-concentration quality control material (Q2) we spiked ammonium acetate 10 mM with 500 pg/mL. The spiked quality control materials were aliquoted and stored at -80°C until analysis. For quality assurance Q1 and Q2 control samples were included in each analytical series for method validation. Stability of the measured compounds was tested by analysing aliquoted and at -80°C freeze Q1 and Q2 solutions.

### Liquid chromatography

**Figure 1:**
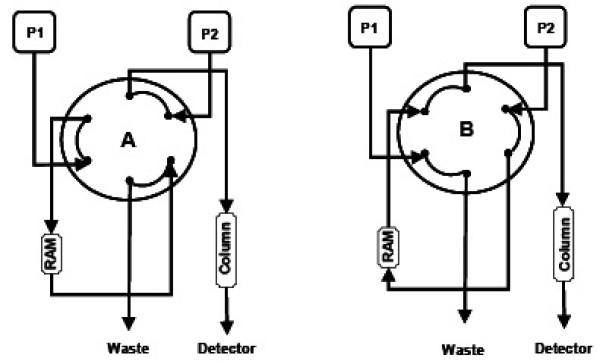
Six-port switching valve arrangement for the clean-up and enrichment step (Valve position A, left side) and the chromatographic separation step (Valve position B, right side). P1 correspond to the isocratic and P2 to the gradient pump.

Liquid chromatography separation was performed on a Hewlett-Packard HP 1100 series HPLC system equipped with a binary gradient pump, an isocratic pump, degasser and Autosampler. The isocratic pump was used to load the 900 μL aliquot sample on a restricted access material (RAM) phase, a LiChrospher RP-18 ADS (25 μm, 25 × 4 mm) from Merck (Darmstadt, Germany) using an ammonium acetate buffer 2 mM (pH 4,6) and methanol (9:1, v/v) as the mobile phase and a flow rate of 0.8 mL/min. The loading of the sample on this RAM phase serves as an enrichment step and to exclude macromolecules such as proteins that were present in the EBC. Next, analytes were transferred in backflush mode through a time controlled six-port valve (Rheodyne) with the LC gradient pump to an analytical HPLC column (Prisma-RP 150 × 2.1 mm, from Thermo). The gradient LC elution condition and the valve switching steps are described in Table 1. All steps were controlled by Analyst 1.3 Software from Perkin Elmer except the isocratic pump. A scheme of the two dimensional column systems is represented in Figure 1.

**Table 1:** Program of time controlled steps for the LC gradient pump and the six-port switching valve.

**Time (min)**	**Flow (mL/min)**	**Solvent A (%)**	**Solvent B (%)**	**Valve Position**
0	0.25	70	30	A
5	0.25	70	30	B
11	0.25	40	60	
12	0.25	0	100	
17	0.25	0	100	
19	0.25	70	30	A
21	0.25	70	30	

Solvent A: Ammonium Acetate buffer 2 mM (pH 4.6)/Acetonitrile (99.5/0.5 v/v)

Solvent B: Ammonium Acetate buffer 2 mM/Acetonitrile/glacial acetic acid (2/97/1; v/v)

### Optimization of online clean-up and enrichment step

A LC-LC column switching method was optimized for the automation of sample clean-up and enrichment for the analysis of inflammatory markers in EBC.

For the automated sample enrichment step a LiChrospher^® ^ADS C18 was used. This is a so-called restricted access material (RAM) phase, where extraction of analytes is based on two chromatographic processes: on one hand reversed phase interactions for the retention of unpolar and middle polar compounds, and on the other hand size exclusion chromatography to avoid macromolecules such as proteins [17]. These macromolecules are eluted with the void volume into the waste. Molecules with a molecular weight up to 15 kDa are able to penetrate the pores and be retained by reversed phase interactions. Also ADS C8 and ADS C4 RAM phases were tested but quantitative retention of all analytes was achieved only by the ADS C18.

The isocratic solvent was optimized to a 2 mM aqueous ammonium acetate solution and methanol (90/10, v/v) to charge the sample onto the RAM ADS 18 phase without analyte losses and with the most clean-up effect from matrix compounds.

After charging and flushing the sample with the isocratic solvent to eliminate macromolecules and polar compounds into the waste, the transfer step to the analytical column can be initiated. Turning the six-port switching valve into position B the analytes can be eluted in backflush mode from the RAM phase with the gradient solvent and transferred to the analytical column for the separation of the analytes (see Figure 1). The starting conditions for the gradient solvent was a composition of 70% solvent A and 30% solvent B (70:30, v/v) being solvent A and solvent B described in Methods.

### Mass spectrometry

A Sciex API 3000 tandem MS system was used for MS-MS detection with an electrospray ion source in the negative ion mode (ESI-). Compound specific mass spectrometer parameters were optimized automatically with the corresponding Sciex Analyst 1.3.1 Software tools by continuous injection of each compound with a syringe pump coupled to the LC/MS/MS system. Source specific parameters that depend on chromatographic conditions were optimized manually. The established ion source parameters were the same for all of the analytes. The applied electrospray needle voltage was – 3500 V and Nitrogen was used as nebulizer and turbo heater gas (500°C) at a pressure of 8 psi each as well as for the collision gas setting at 10 instrument units. The curtain gas was set to 8 psi. MRM (multiple reaction monitoring) mode was chosen to perform the MS-MS detection. MRM mode allows a simultaneous registration of all MS-MS transitions at a scan time of 150 ms for each fragmentation. At the used ESI negative mode, the selected precursor ions at the first quadrupole for all analytes were [M-H]^-^. The product ion fragments selected were with the maximum intensities for all the analytes ensuring maximum of sensitivity. The substance specific mass spectrometer conditions for each compound are listed in Table 2 and were performed with continuous flow injections of standard solutions of all analytes with a coupled syringe pump system to the Sciex API 3000 LC/MS/MS system. So it was possible to find the most specific and intense parent-daughter ion transitions for each compound for the tandem MS detection (see Table 2).

**Table 2:** Compound specific mass spectrometer conditions.

**Analyte**	**Ret. time (min)**	**Precursor ion**	**Product ion**	**DP**	**FP**	**CE**	**CXP**
8-iso-PGF 2alfa	12.3	353.2	309.2	-66	-310	-26	-17
11-β-PGF 2alfa	12.4	353.2	309.2	-66	-310	-26	-17
PGF 2alfa	12.7	353.2	309.2	-66	-310	-26	-17
PGE 2	13.0	351.2	315.2	-36	-170	-16	-15
PGD 2	13.4	351.2	315.2	-36	-170	-16	-15
13,14-dihydro-15-keto-PGE 2	13.9	351.2	333.3	-26	-150	-16	-15
13,14-dihydro-15-keto-PGD 2	14.4	351.2	333.3	-26	-150	-16	-15
LTE 4	14.5	438.2	333.2	-14	-70	-24	-17
Delta 12-PGJ 2	14.7	333.2	315.1	-36	-200	-12	-17
PGJ 2	14.8	333.2	315.1	-36	-200	-12	-17
LTB 4	15.3	335.2	195.2	-31	-150	-16	-15
15-desoxy-delta12,14-PGJ 2	16.2	315.1	271.1	-45	-210	-16	-15

Declustering and Focusing Potential as well as Collision Potential are expressed in Volts (V)

**Table 3:** Reliability data of the method for the determination of eicosanoids in exhaled breath condensate.

Analyte	Intra-day precision		Inter-day precision		Accuracy		Calibration (Y = ax+b)	
	Q1	Q2	Q1	Q2	relative recovery (%)		a (x10e-3)	b (x10e-3)
	RSD(%)	RSD(%)	RSD(%)	RSD(%)	mean	range		
8-iso-PGF 2alfa	5	5	16	11	94	88–107	0,098	2,21
11-β-PGF 2alfa	4	2	10	8	102	97–108	0,084	0,358
PGF 2alfa	2	2	13	8	102	99–104	0,254	0,314
PGE 2	4	2	5	6	99	96–107	0,628	1,92
PGD 2	4	2	4	6	102	94–112	0,345	0,089
13,14-dihydro-15-keto-PGE 2	2	2	8	8	94	90–100	1,46	-0,245
13,14-dihydro-15-keto-PGD 2	2	3	9	8	104	99–110	1,04	4,88
LTE 4	6	4	10	7	115	96–133	0,238	1,3
Delta 12-PGJ 2	5	5	13	11	105	98–120	1,09	1,2
PGJ 2	5	6	4	8	110	102–119	0,213	1,17
LTB 4	5	5	9	11	110	97–117	0,19	1,4
15-desoxy-delta12,14-PGJ 2	5	5	16	12	159	135–179	0,739	5,46

Q1 and Q2 represents the low and the high concentration level respectively, with 50 pg/mL and 500 pg/mL each analyte.

## Results and discussion

### Optimization of enrichment and chromatography

**Figure 2:**
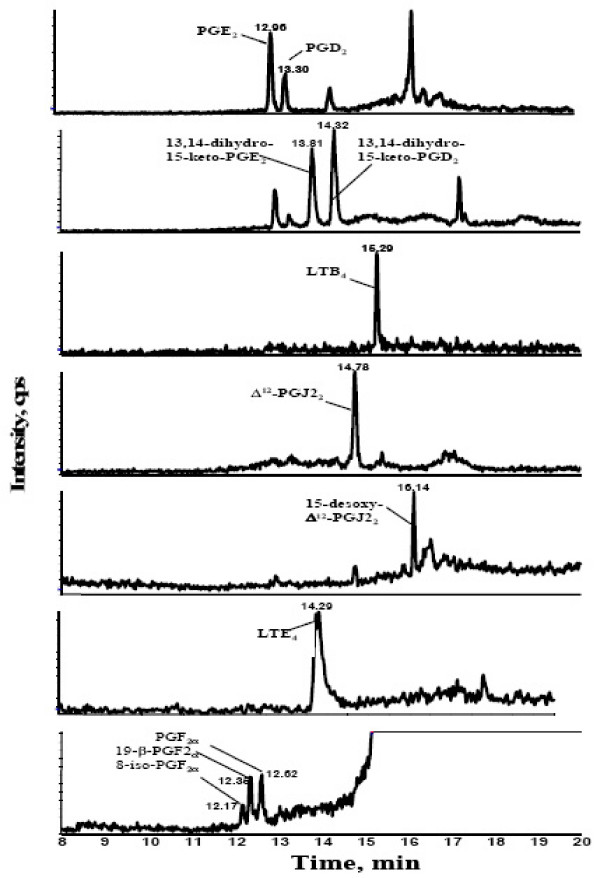
chromatogram of a spiked EBC sample as an example.

Increasing the fraction of solvent B as shown in Table 1 the analytes can be eluted from the RAM Phase and separated at the analytical column before detection. After all analytes were eluted from the analytical column, both RAM and analytical column were washing with 100% solvent B before reconditioning for the next run. Optimization of the chromatographic separation of the analytes was necessary to distinguish some of the structural isomers of prostaglandins, which resulted in the same parent-daughter ion transitions. The whole analytical run time, including the recondition step of the column for the next injection, was 21 min. Figure 2 represents an example of a chromatogram of spiked EBC with each analyte.

### Mass spectrometry and internal standardization

An enhanced detector response for the analytes was achieved by using a 2 mM ammoniumacetate solvent as mobile phase in contrast to water or higher concentrated ammoniumacetate buffer. This is probably due to an optimized ionisation condition at the ion source for these substances. Only the PGF_2α _derivatives have a 10–25 % improved response using bi-distilled water as mobile phase. Trying to cover most of the analytes with the highest possible response 2 mM ammoniumacetate buffer was selected as mobile phase.

**Figure 4:**
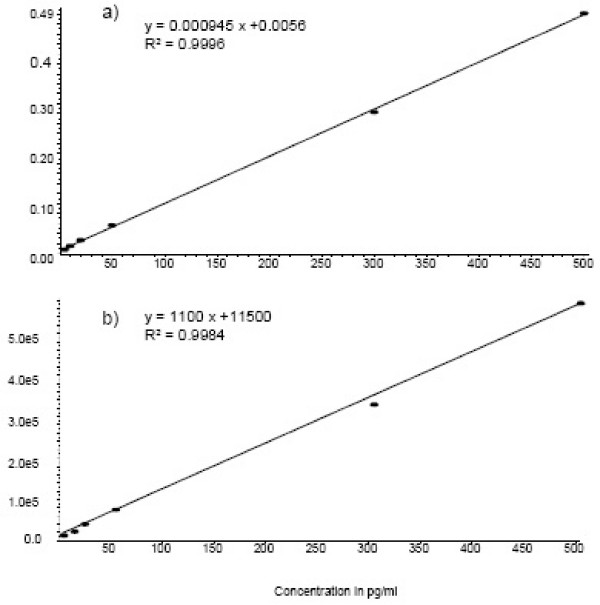
a) Calibration curve of 13,14-dihydro-15-keto-PGD2 with PGE2-d4 as internal standard and b) without internal standard.

Using the area counts of [^2^H_4_] LTB_4 _and [^2^H_4_] PGE_2 _as correctional factor of all other leukotrienes and prostaglandins, respectively, shows better correlation coefficient of the calibration curve at the linear regression than that renouncing the application of these internal standards to the homologous analytes (Figure 4). Even as these used internal standards have just similar chromatographic behaviour as to the applied different analytes, so it was possible to show a higher correlation applying each internal standard to the corresponded group of substances than without internal standard.

### Reliability of the method

Comparing calibrations achieved with analytes spiked in 2 mM ammoniumacetate buffer and pooled EBC it was possible to demonstrate no matrix influence to the slope and linearity of the calibration curve. Due to the low content of matrix compounds in EBC in contrast to other matrix such as urine or plasma which could influence the response of the analytes in question, no matrix effect was observed as expected. EBC is mainly formed by water vapour which contains non-volatile compounds in the aerosol particles carried away during breathing. Pooled EBC was used as representative matrix for individual gained EBC.

Calibration curves with spiked EBC are congruent with the curves performed in ammonium acetate buffer 10 mM. Thus, calibration curves were obtained by spiking increased amounts of analytes in 2 mM ammoniumacetate and in pooled EBC. All calibration curves obtained in the range from 10 to 500 pg/mL were linear (Figure 4 as an example and see Table 3) and produced linear correlation coefficients greater than 0.99.

### Precision and accuracy

The intraday repeatability was addressed by analysing Q1 and Q2 ten times in a row and on six different days resulting in a relative standard deviation for all parameters in the range from 2–6% for both levels of concentration. The relative standard deviation of the between-day repeatability for the Q1 and Q2 level ranged from 4–16% and from 6–12% respectively.

Accuracy was obtained from the ratio of the calculated and the nominal amount spiked for both mentioned concentration levels measured ten times in a row. At the Q1 and Q2 level accuracies for all analytes except 15-deoxy-Δ^12,14^-PGJ_2 _ranged from 93–120% and from 88–133% respectively. For the mentioned 15-desoxy-Δ^12,14^-PGJ_2 _mean accuracy was about 150%, resulting in an overestimation for the calculated concentration. This could be due to the lack of an appropriate internal standard for the mentioned substance in contrast to the other analytes where the used internal standard seems to mirror the behaviour of the assigned compounds. Another possible reason could be a positive matrix effect for this analyte where other matrix compound could enhance the ionization at the source for the analyte in question. The data showing the reliability of the method is presented on Table 3.

### Limit of detection and quantification

The limits of detection (LOD), defined as a signal to noise ratio of three for the registered fragment ions, were estimated to be about 5 pg/mL.

The limits of quantification (LOQ) defined as a signal to noise ratio of six for the registered fragment ions, were estimated to be about 10 pg/mL.

### Stability of analytes

No decreases in the concentration of the compounds were observed over a period of about 8 weeks stored at -80°C.

### General considerations

In the literature, measurements of PGE_2 _and PGF_2α _are increased in exhaled breath condensate from patients with COPD.

Leukotrienes were detected in EBC samples from asthmatic and healthy subjects by both, immunoassay and GC/MS [3,7]. The median exhaled concentrations of LTD_4_, LTE_4 _and LTB_4 _in asthmatic individuals (adults and childrens) were increased compared with those of healthy adults and children respectively [7].

Some studies were conducted to determine 8-isoprostane in EBC of asthmatic patients [12], of children with asthma exacerbations [11], subjects with COPD [12] and patients with cystic fibrosis [13]. Carpagnano et al. [12] found an increased mean concentration of 8-isoprostane in EBC samples of COPD patients compared to healthy subjects.

All these studies deal with determinations of inflammatory markers which serve as biological marker, differentiating between increased concentration levels in patients from lower endogenous concentration levels in EBC in healthy subjects.

Most of the data found in the literature were determinations made by ELISA or RIA, where antibodies cross reactivity should be considered. There is limited knowledge about the reliability of enzyme immunoassay kit to determine inflammatory marker in EBC. Il'Yasova et al [18] report about a method comparison of the determination of an isoprostane derivative in urine using GC/MS and ELISA. With the ELISA a 30-fold overestimation in contrast to the GC/MS was obtained for this parameter in urine.

It is not possible to determine simultaneously different inflammatory markers in one run with ELISA technology, whereas other advantages such as cost effectiveness and high throughput analysis should be noted for ELISA. For research purposes it could be important to monitor different parameters simultaneous to can relate different markers or a class of substance to different diseases. However due to the small sample volumes of EBC obtained, this advantage of determine several substances in one run should be emphasized.

In contrast to the GC/MS methods, LC/MS has the advantage, that derivatization procedures and corresponding sample pre-treatment for non volatile compounds is not required, therefore avoiding more sources of errors. The specificity of the MS detection ensures an unequivocal determination of the analysed substances.

## Conclusion

The mostly applied quantification method for the analyses of eicosanoids in EBC was commercially available enzyme linked immunoassays, which is very sensitive, but lack in specificity and detection related to structural information such as mass spectrometry.

Our developed method allows for a sensitive, specific and reliable determination of leukotriens and prostaglandins in EBC, thus avoiding sources of errors due to the application of automated sample pre-treatment steps. With the method presented here it is possible to detect prostaglandins and leukotriens derivatives simultaneously up to a LOQ of 10 pg/mL respectively and could be very useful for the findings of new biomarkers of pulmonary diseases or even to apply other methodologies for risk assessment such as metabonomics. Application of such methods could be help to the breakthrough of assessments of pulmonary diseases using exhaled breath condensate as an easy gained sample matrix for diagnostics.

In this context a critical review about the utility of EBC for pulmonary investigators and clinicians is described by Effros et al. [19].

## Competing interests

The author(s) declare that they have no competing interests.

## Authors' contributions

LMGR and AKM carried out the method development. AML, TG and TK participated in the conceiving of the study and helped to draft the manuscript.

## References

[B1] Kharitonov SA, Barnes PJ (2001). Exhaled markers of pulmonary disease. Am J Respir Crit Care Med.

[B2] Barnes PJ, Chung KF, Page CP (1998). Inflammatory mediators of asthma: an update. Pharmacol Rev.

[B3] Carpagnano GE, Barnes PJ, Geddes DM, Hodson ME, Kharitonov SA (2003). Increased leukotriene B4 and interleukin-6 in exhaled breath condensate in cystic fibrosis. Am J Respir Crit Care Med.

[B4] Repine JE, Bast A, Lankhorst I (1997). Oxidative stress in chronic obstructive pulmonary disease. Oxidative Stress Study Group. Am J Respir Crit Care Med.

[B5] Kharitonov SA, Barnes PJ (2001). Exhaled markers of inflammation. Curr Opin Allergy Clin Immunol.

[B6] Pavord ID, Tattersfield AE (1995). Bronchoprotective role for endogenous prostaglandin E2. Lancet.

[B7] Cap P, Chladek J, Pehal F, Maly M, Petru V, Barnes PJ, Montuschi P (2004). Gas chromatography/mass spectrometry analysis of exhaled leukotrienes in asthmatic patients. Thorax.

[B8] Morrow JD, Roberts LJ (1997). The isoprostanes: unique bioactive products of lipid peroxidation. Prog Lipid Res.

[B9] Mori TA, Dunstan DW, Burke V, Croft KD, Rivera JH, Beilin LJ, Puddey IB (1999). Effect of dietary fish and exercise training on urinary F2-isoprostane excretion in non-insulin-dependent diabetic patients. Metabolism.

[B10] Montuschi P, Corradi M, Ciabattoni G, Nightingale J, Kharitonov SA, Barnes PJ (1999). Increased 8-isoprostane, a marker of oxidative stress, in exhaled condensate of asthma patients. Am J Respir Crit Care Med.

[B11] Baraldi E, Carraro S, Alinovi R, Pesci A, Ghiro L, Bodini A, Piacentini G, Zacchello F, Zanconato S (2003). Cysteinyl leukotrienes and 8-isoprostane in exhaled breath condensate of children with asthma exacerbations. Thorax.

[B12] Carpagnano GE, Kharitonov SA, Foschino-Barbaro MP, Resta O, Gramiccioni E, Barnes PJ (2004). Supplementary oxygen in healthy subjects and those with COPD increases oxidative stress and airway inflammation. Thorax.

[B13] Montuschi P, Kharitonov SA, Ciabattoni G, Corradi M, Van Rensen L, Geddes DM, Hodson ME, Barnes PJ (2000). Exhaled 8-isoprostane as a new non-invasive biomarker of oxidative stress in cystic fibrosis. Thorax.

[B14] Montuschi P, Martello S, Felli M, Mondino C, Chiarotti M (2004). Ion trap liquid chromatography/tandem mass spectrometry analysis of leukotriene B4 in exhaled breath condensate. Rapid Commun Mass Spectrom.

[B15] Montuschi P, Ragazzoni E, Valente S, Corbo G, Mondino C, Ciappi G, Ciabattoni G (2003). Validation of leukotriene B4 measurements in exhaled breath condensate. Inflamm Res.

[B16] Mutlu GM, Garey KW, Robbins RA, Danziger LH, Rubinstein I (2001). Collection and analysis of exhaled breath condensate in humans. Am J Respir Crit Care Med.

[B17] van der Hoeven RAM, Hofte AJP, Frenay M, Irth H, Tjaden UR, Van der Greef J, Rudolphi A, Boos KS, Marko-Varga G, Edholm LE (1997). Liquid chromatography-mass spectrometry with on-line solid-phase extraction by a restricted-access C18 precolumn for direct plasma and urine injection. J Chrom A.

[B18] Il'Yasova D, Morrow JD, Ivanova A, Wagenknecht LE (2004). Epidemiological marker for oxidant status: comparison of the ELISA and the gas chromatography/mass spectrometry assay for urine 2,3-dinor-5,6-dihydro-15-F2t-isoprostane. Ann Epidemiol.

[B19] Effros RM, Su J, Casaburi R, Shaker R, Biller J, Dunning M (2005). Utility of exhaled breath condensates in chronic obstructive pulmonary disease: a critical review. Curr Opin Pulm Med.

